# Research progress of nano-based drug delivery systems based on stimuli-responsive materials for the treatment of lung diseases

**DOI:** 10.3389/fbioe.2025.1644007

**Published:** 2025-07-31

**Authors:** Wenqiang Li, Qian Huang, Mei Li, Youli Wen, Zhao Chen, Yuting Fan, Chen Shen, Chen Gong, Yao Luo, Zhiping Deng

**Affiliations:** ^1^ Department of Pulmonary and Critical Care Medicine, Zigong First People’s Hospital, Zigong, China; ^2^ Department of Pulmonary and Critical Care Medicine, Dazhou Dachuan District People’s Hospital (Dazhou Third People’s Hospital), Dazhou, China; ^3^ Department of Laboratory Medicine, West China Hospital, Sichuan Clinical Research Center for Laboratory Medicine, Sichuan University, Chengdu, China; ^4^ Department of Clinical Medicine, North Sichuan Medical College, Nanchong, China; ^5^ Department of Oncology, Tongji Hospital, Tongji Medical College, Huazhong University of Science and Technology, Wuhan, China

**Keywords:** stimuli-responsive materials, NDDSs, internal and external environments, lung diseases, treatment

## Abstract

Since the lungs are directly connected to the external environment and have a rich blood supply, they are susceptible to damage and tumor growth. However, the pharmacokinetics of traditional drugs in the lungs are limited when administered orally or intravenously, posing challenges for clinical treatment. Compared to traditional drug delivery methods, nano-based drug delivery systems (NDDSs) have the advantages of high drug loading capacity, strong targeting, low cellular toxicity, and extended circulation time in the blood. Stimuli-responsive materials, often referred to as “smart” materials, are a class of functional materials that can change their properties in response to various stimuli in both internal and external environments. Therefore, stimuli-responsive materials have gradually become promising candidates for NDDSs. To date, many stimuli-responsive NDDSs have been developed for treating lung diseases. Our review primarily summarizes the novel NDDSs that have emerged in recent years for treating common benign and malignant lesions in the lungs, based on stimuli-responsive materials. Finally, we discussed the existing issues in stimuli-responsive NDDSs and looked forward to their future development prospects.

## Graphical Abstract

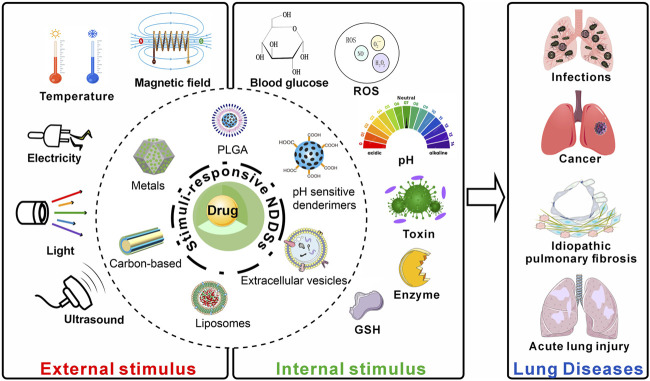

## 1 Introduction

Lung diseases encompass a variety of conditions affecting the lungs, which can arise from multiple factors, including infections, environmental influences, genetic predispositions, and others (Odendaal et al., 2024; Wang T. et al., 2024). Among these, the inhalation of external microorganisms and the dysregulation of the lungs’ internal microbiota are important factors leading to lung infections (Li R. et al., 2024; Natalini et al., 2023). Lung cancer (LC) is typically classified into small cell lung cancer (SCLC) and non-small cell lung cancer (NSCLC), with NSCLC accounting for more than 85% of all cases (Leiter et al., 2023). As environmental pollution, food safety issues, and exposure to volatile chemicals in daily life increase, the risk of lung problems is also on the rise (Liang et al., 2023; Zhu et al., 2023).

Due to the complexity of the respiratory system and the challenges posed by traditional treatment methods, there is an urgent need to develop innovative treatment strategies. In recent years, with the rapid advancement of science and technology, nanomaterials have provided a viable platform for drug loading and delivery. Nano-based drug delivery systems (NDDSs) are considered an effective approach for treating lung diseases because they can overcome the limitations of traditional treatments (Nayak et al., 2025). Stimuli-responsive nanomaterials can experience controllable changes in their physicochemical properties when exposed to various environmental conditions, including physiological stimuli such as pH, enzymes, and redox potential, as well as external energy stimuli like light, magnetic fields, and ultrasound (Wang et al., 2022; Wei et al., 2023). Compared to traditional drug delivery nanosystems, stimuli-responsive NDDSs offer many advantages, such as high sensitivity, broad applicability to various diseases, and diverse functionalities (Wei et al., 2023). In particular, the use of multi-stimuli responsive strategies to achieve sequential or cascade drug delivery has attracted significant attention. In recent years, researchers have reported a series of stimuli-responsive NDDSs for lung diseases, including polymer vesicles, dendrimers, hydrogel capsules, metal nanoparticles (NPs), and lipid NPs (Su et al., 2023; Huang et al., 2022; Fischer et al., 2023; Li H. et al., 2024; Reczyńska et al., 2020).

**FIGURE 1:**
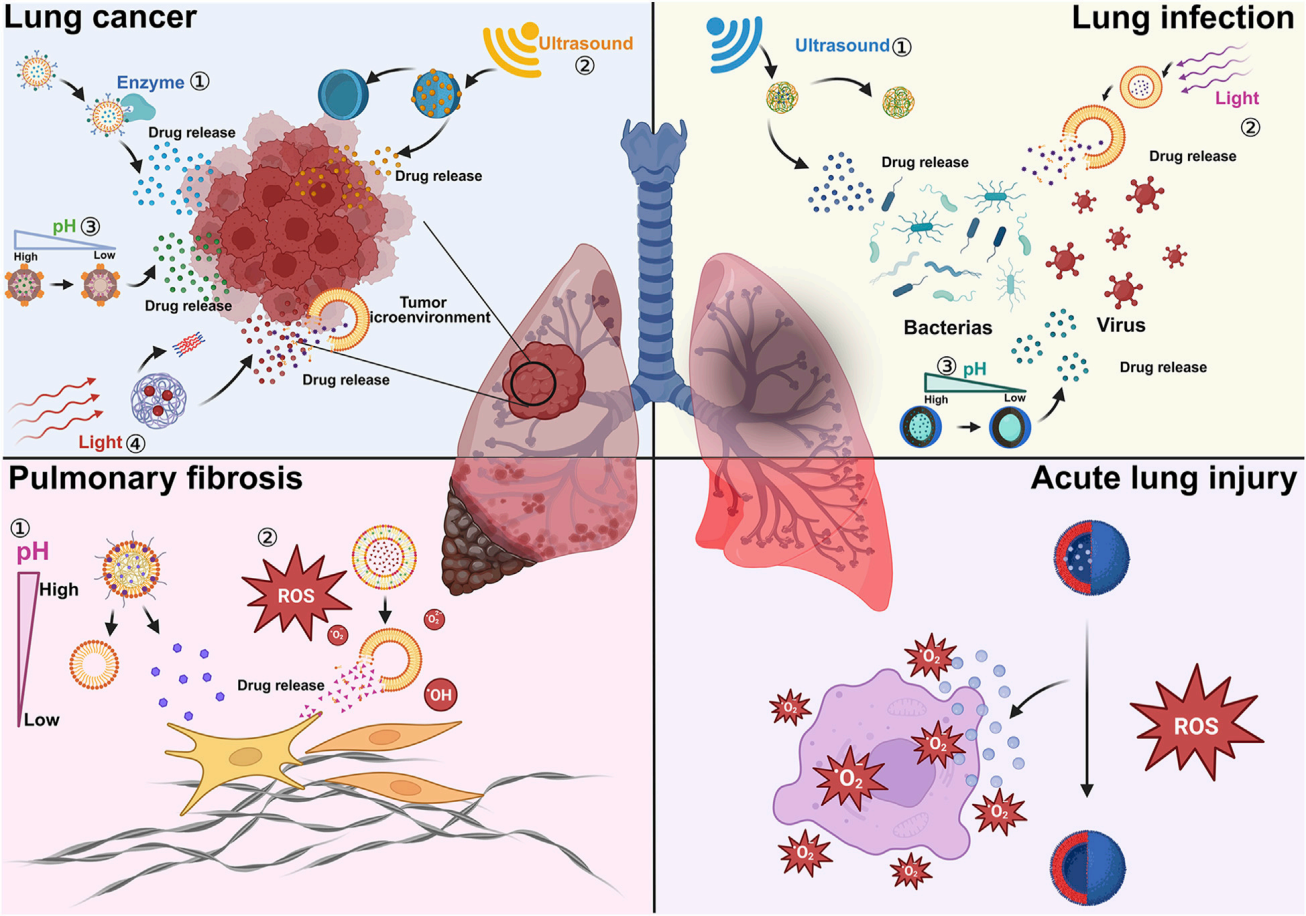
Stimuli-responsive nanosystems for the treatment of lung diseases.

Our review primarily focuses on summarizing the stimuli-responsive NDDSs that have been developed recently for treating lung infections, LC, idiopathic pulmonary fibrosis (IPF), and acute lung injury (ALI) (Figure 1). Following that, the review discusses the challenges in the clinical translation of these NDDSs and looks ahead to their future application prospects.

## 2 An introduction to stimuli-responsive NDDSs

The internal microenvironment of the human body is complex, and the pathological microenvironment affects the occurrence and development of diseases. Within this microenvironment, the pH levels of pathological sites, such as infections, inflammation, and tumors, differ from those of normal tissues (Feng et al., 2024). When the pH changes, stimuli-responsive NDDSs can respond by expanding, contracting, or cleaving their functional groups, thereby releasing the encapsulated drugs (Lv et al., 2024; Fan et al., 2021; Wang S. et al., 2024). In addition, enzymes play an important role in the human body. The assembled responsive NDDSs can be transformed by enzymes into detachable structures, leading to a reduction in size that facilitates penetration into diseased and injured tissues, as well as the release of encapsulated drugs (Chen et al., 2024). External stimuli, such as light, ultrasound, magnetic fields, temperature, and radiation, offer more precise spatiotemporal adjustability and can be added or removed based on the treatment needs (Wei et al., 2023; Ding et al., 2024). Many pulmonary diseases (such as chronic obstructive pulmonary disease (COPD), cystic fibrosis, and lung infections) are characterized by excessive mucus production, which can hinder the delivery of drugs to the target site. Stimuli-responsive NDDSs can be designed with specific trigger mechanisms (such as pH sensitivity, enzyme sensitivity, or temperature sensitivity, etc.) to release drugs upon encountering mucus, thereby effectively penetrating the mucus barrier (Yu et al., 2025; Wang et al., 2024a; Wang et al., 2024b). Immunotherapy has emerged as a significant tumor treatment strategy following the clinical application of targeted therapies. However, due to tumor heterogeneity and the low immunogenicity of the tumor microenvironment (TME), only a small fraction of tumors are sensitive to immune checkpoint inhibitors. Therefore, it is crucial to develop nanocarriers that selectively activate various innate immune pathways, enhance innate immune responses, and reduce side effects. In particular, the use of nanocarriers for targeted drug delivery, combined with the research on environmentally responsive NPs, offers new perspectives for the immunotherapy of NSCLC (Li Y. et al., 2024; Li M. et al., 2024).

In recent years, stimuli-responsive nanocarriers have experienced rapid development in areas such as cancer treatment, anti-inflammatory therapy, and antimicrobial applications (Zhang et al., 2023; Yu et al., 2024; Peng et al., 2024). Currently, research on stimuli-responsive nanocarriers is primarily focused on synthetic polymers and biomaterials. Compared to synthetic polymers, biomaterials and their derivatives are gaining increasing attention due to their unique advantages, such as biodegradability, biocompatibility, natural abundance, and unique chemical structures (Datta et al., 2020). In particular, stimuli-responsive NDDSs based on biomaterials have the potential to address issues such as poor water solubility, less precise targeting, limited dispersibility, and high toxicity, which are difficult to overcome in traditional drug delivery systems. However, various types of stimuli-responsive NDDSs exhibit distinct advantages and disadvantages (Table 1). These nanocomposites offer new options for effective disease treatment.

**TABLE 1:** Advantages and disadvantages of different types of stimulus-responsive NDDSs.

Stimuli-responsive NDDSs	Advantages	Disadvantages	References
pH-responsive NDDSs	Better at penetrating the biomembrane	Can be easily recognized by opsonins in the plasma and thus cleared	Yan and Ding (2020), Chen et al. (2023)
Enzyme-responsive NDDSs	More suitable for the complex TME.	It lacks the specificity required for targeted cancer immunotherapy; nonspecific enzyme activity can cause off-target effects; and it is difficult to precisely control the kinetics of enzymatic reactions	Peng et al. (2022), Li et al. (2020a), Shahriari et al. (2019)
ROS-responsive NDDSs	Suitable for combination with tumor-targeted therapy	Lacks selectivity because the redox conditions in both healthy and pathological tissues can cause the unintended release of the immunotherapy agent	Li et al. (2020b), Yang and Sun (2022), Li et al. (2020c)
Ultrasound-responsive NDDSs	Can control the timing of drug release and allow for repeated dosing	Due to the limitations in penetration capability, they are difficult to achieve precise spatial localization	Xiu et al. (2023), Cheng et al. (2021), Zhou et al. (2020)
Light-responsive NDDSs	Can remotely trigger drug release in an on-off manner	Affected by tissue depth, the effective range is limited	Kang et al. (2023), Chen and Zhao (2018)

## 3 Stimuli-responsive NDDSs for anti-lung infection

Lung infection refers to the inflammatory response caused by the invasion of pathogens such as bacteria, viruses, and fungi into the lungs (Pettigrew et al., 2021). For lower respiratory tract infections caused by bacteria, systemic antibiotic administration is commonly used in clinical treatment (Lalmohamed et al., 2024). However, systemic drug administration has characteristics such as low drug utilization, significant toxicity to surrounding tissues, and a tendency to induce bacterial resistance (Pani and Mohapatra, 2024). Moreover, due to the unique pathological environment of the lower respiratory tract, it is more challenging to efficiently deliver drugs to eliminate bacteria within the lung mucous layer and biofilms. These NDDSs have been extensively validated for safety and efficacy in animal models of pulmonary infections caused by *Pseudomonas aeruginosa* (*PA*) and *Staphylococcus aureus* (*S. aureus*).

*PA* is an important pathogen in respiratory tract infections, particularly in the lower respiratory tract (Weimann et al., 2024). The cell membrane of *PA* can hinder drug penetration and the phagocytosis of immune cells. The bacterium produces virulence factors that inhibit the host’s immune response, leading to antibiotic tolerance and immune evasion (Du Toit, 2024). A responsive NDDS prepared from dimethylmaleic anhydride (DA) and azithromycin (AZI), epsilon-poly (l-lysine) (DA-AZI NPs). Acidic conditions can promote the penetration of DA-AZI NPs through mucus and biofilms, ultimately allowing the carried antibiotics to better exert effects against *PA* infections (Li R. et al., 2024). Curcumin (Cur) and dimethylmaleic anhydride (DA) were modified with anti-CD54 to form anti-CD54@Cur-DA NPs. The release of Cur from Cur-DA NPs at acidic pH results in charge reversal and size reduction, which facilitates increased penetration of *PA* biofilms and enhances the antibacterial effects of the antibiotics (Chen et al., 2023). In addition, some stimulus-responsive NDDSs rely on external physical stimuli. In recent years, there have been many breakthroughs in ultrasound-stimulated (US) responsive NDDSs. Chlorin e6 (Ce6) and metronidazole (MNZ) were incorporated into liposome (Lip) NPs encapsulating perfluoropentane (PFP), ultimately forming PLCM NPs. Under US, Ce6 and MNZ are more easily released from PLCM NPs, which induces the formation of numerous pores in the *PA* cell membrane, thus enhancing the antibiotic’s bactericidal effect (Xiu et al., 2024). Another team utilized Fe_3_O_4_ NPs and the antibiotic piperacillin (Pip) to construct ultrasound-responsive catalytic microbubbles (MB-Pip). Under ultrasound, MB-Pip disrupt the biofilm structure through mechanical effects and release Fe_3_O_4_ NPs, which degrade the extracellular polymeric substance (EPS) matrix. This results in physical and chemical biofilm disruption, enhancing the drug’s penetration and antibacterial performance (Xiu et al., 2023). A human umbilical cord mesenchymal stem cell membrane (MSCm) modified with the bacterial targeting peptide UBI29-41, ZIF-8 metal-organic framework, and organic silica NPs makes up PMZMU. Upon activation by ultrasound, PMZMU can eliminate bacterial biofilms, as confirmed in a *PA*-induced mouse pneumonia model study (Huang J. et al., 2024). In addition to ultrasound, new research results have also been reported on responsive NDDSs activated by physical radiation. The photosensitizer black phosphorus quantum dots (BPQDs) and the antibiotic amikacin (AM) were loaded into a biomimetic liposome (AB@LRM) constructed by fusing red blood cell membranes and macrophage cell membranes, resulting in the synthesis of AB@LRM NPs. Under near-infrared (NIR) radiation, BPQDs generate reactive oxygen species (ROS) and heat, which enhance the thermal sensitivity of *PA* cell membranes, disrupt their structure, and improve the antibacterial effect (Liu et al., 2024). In addition to directly damaging the cell membrane, reversing the resistance conditions of dormant *PA* biofilms for infection treatment is also an effective approach. Composed of maltahexose (GP), catalase, and gallium ions, the nanosystem (MCPGaGP) is capable of awakening the metabolism of *PA* within the biofilm. Finally, by activating the secretion of more iron ion uptake channels in *PA*, this nanosystem enhances the self-destructive absorption of the nutrient iron-gallium analog (He et al., 2024).

Among the pathogens causing lung infections, *S. aureus* is also a key bacterium of concern for clinical practitioners (Zaghen et al., 2023). The nanosystem consists of D-alanine functionalized gold NPs (DAu NPs) encapsulated by a macrophage membrane (MM) coating, known as MM@DAu NPs. After exposure to NIR, the accumulation of DAu NPs around the bacteria within the cells induces localized hyperthermia, enabling precise eradication of *S. aureus* within lung cells (Xiong et al., 2024). Pure ciprofloxacin NPs (NanoCip) integrated with PBP2a antibody-modified membrane nanovesicles (AMVs) form a novel biomimetic nanomedicine known as AMV@NanoCip. After US, this NDDS exhibits significant *S. aureus*-targeting affinity in both *in vitro* and *in vivo* models, thereby greatly enhancing its antibacterial activity (Ding et al., 2024). Li et al. developed a novel nanodelivery system by combining bovine serum albumin, polydopamine, and Ag_2_O_2_, known as Ag_2_O_2_@BP-MT@MM. This system effectively eliminates the activity of metallo-*β*-lactamase (MBL) by replacing the Zn^2+^ cofactor in MBL with Ag^+^, thereby demonstrating effective bactericidal properties (Li H. et al., 2024).

## 4 Stimuli-responsive NDDSs for anti-LC

LC is one of the most lethal cancers globally, with NSCLC being the most common type, but it has a low overall survival rate (Tammemägi et al., 2024). Due to the characteristics of the TME, stimulus-responsive polymers specifically targeting the TME have been widely used to prepare intelligent nanocarriers for targeted delivery of therapeutic agents and diagnostic reagents to tumor tissues. Compared to traditional NDDSs, stimulus-responsive NDDSs have many advantages, such as high sensitivity, broad applicability across different tumors, multifunctionality, and improved biosafety.

Matrix metalloproteinases (MMPs) belong to the family of zinc-dependent endopeptidases and are one of the commonly overexpressed enzymes in the TME. The research team utilized MMP2-responsive peptides to construct a complex conjugated with miR-126-3p (MAIN) and further disguised it with red blood cell (RBC) membranes (named REMAIN), targeting the overexpressed MMP2 in the TME. REMAIN can effectively transduce miRNA into LC cells, releasing the miRNA in response to MMP2, and ultimately induce apoptosis in lung adenocarcinoma cells (Liang et al., 2022). In addition, micro-NPs (GSC) made from silk fibroin (SF) and gelatin were prepared for MMP9 in the TME, which were fabricated to load paclitaxel (PTX@GSC), PD-L1 antibodies (αPD-L1@GSC), and PD-L1 small interfering RNA (siPD-L1@GSC) respectively. These stimulus-responsive NDDSs require prolonged exposure to a high MMP-9 environment to release the drugs, which increases the specificity and targeting of the anti-tumor effects (Gou et al., 2023). To enhance the immune response to NSCLC, a team of scientists in China has developed a folic acid-modified liposomal nano-bubble for precise delivery of PFH, STAT3 siRNA, and Fe_3_O_4_ to the TME. These nano-bubbles can undergo phase transition and release Fe_3_O_4_ under low-intensity focused ultrasound (LIFU), which can activate the IRF5 signaling pathway. This ultimately promotes the transformation of M2-type macrophages into M1-type while simultaneously inhibiting the polarization of M2-type macrophages (Li P. et al., 2024).

Epidermal growth factor receptor (EGFR) is the most common mutation site in NSCLC, and there are currently several targeted drugs available for this mutation (Pan et al., 2024). Recent studies have shown that stimulus-responsive NDDSs play a positive role in targeted therapy. A NDDS composed of hyaluronic acid (HA), boron dipyrromethene (BPY), adamantane, the peptide sequence GFLG, and gefitinib (Gef) is termed HA-BPY-GEF-NPs. HA-BPY-GEF-NPs generate ROS upon NIR stimulation, which directly eliminate tumor cells. Subsequently, under the stimulation of enzyme (cathepsin B), this nanodrug delivery system releases Gef in a targeted manner (Huang Q. et al., 2024). Another research team utilized dendrimer-based NPs to target the co-delivery of Gef and YAP gene-silencing siRNA (YAP-siRNA). In a reducting environment, this NDDS releases Gef and YAP-siRNA. Additionally, under laser irradiation, the NPs can produce strong antitumor effects without causing significant toxicity (Huang et al., 2022).

LC is prone to metastasis, which is closely associated with poor prognosis. Therefore, recent studies have focused on exploring NDDSs aimed at blocking metastasis (Li et al., 2021; Fan et al., 2023). A recent study reported a pH-responsive NDDS based on DNA tetrahedral framework nucleic acids for the simultaneous delivery of immunomodulatory CpG oligonucleotides and PD-L1-targeting antagonistic DNA aptamers, which effectively treats lung metastatic cancer (Fan et al., 2023).

## 5 Stimulus-responsive NDDSs for IPF

IPF is a chronic, progressive, and highly lethal lung disease with a short average survival period following diagnosis (Wijsenbeek and Cottin, 2020). It is characterized by damage to alveolar epithelial cells (AECs), accompanied by enhanced activation/stimulation of fibroblasts and myofibroblasts, leading to the accumulation of extracellular matrix (ECM) in the alveolar walls (Podolanczuk et al., 2023). Mesenchymal stem cells (MSCs) transplantation has been proven to be an effective treatment for IPF (Averyanov et al., 2020). However, the poor microenvironment caused by inflammation, fibrosis, and high levels of ROS in IPF leads to low survival rates and poor function of transplanted MSCs, significantly impacting their therapeutic efficacy. Additionally, the mechanism of action for MSCs in treating IPF is not yet fully understood, greatly limiting the clinical translation of stem cell therapy. Therefore, removing excess intracellular ROS and imaging tracking have become important strategies to protect MSCs. Metal NPs are widely used in biomedical applications related to pulmonary fibrosis. Their antioxidant properties and ability to enhance imaging functions have shown great potential in treating IPF and in sensors for detecting specific biomarkers of IPF. By combining zinc ions and 7,8-dihydroxyflavone with fasudil (a ROCK inhibitor), ZDFPR NPs can reduce mechanical tension in type II alveolar epithelial cells (AEC II) and disrupt the ROCK signaling pathway, thereby reducing the formation of ROS after aerosol inhalation (Li X. N. et al., 2024). By encapsulating copper-based nanozymes (CuxO NPs) and gold NPs (AuNPs) in oxidation-sensitive dextran (Oxi-Dex), ROS-responsive nanocomposites (RSNPs) were successfully created. This nanosystem can eliminate ROS and enable long-term CT imaging tracking of MSCs, thereby allowing a deeper understanding of the cellular therapy mechanisms (Li et al., 2023). Additionally, a pH-sensitive Au nanotracer (CPP-PSD@Au) was prepared by combining AuNPs with sulfonamide-based polymers (PSD) and cell-penetrating peptides (CPP). It can monitor MSCs via CT imaging for up to 35 days after transplantation into the lungs of IPF mice (Yu et al., 2021). Bao et al. prepared a non-viral bifunctional nanocarrier using AuNPs stabilized by protamine sulfate, which can be used for simultaneous IPF treatment and the monitoring the biological behavior of MSCs (Bao et al., 2022). Furthermore, the team subsequently developed a tri-metallic nanocarrier (TBNCs) with similar functionality, using protamine sulfate and three metals: gold (Au), platinum (Pt), and cobalt (Co) (Bao et al., 2024).

## 6 Stimulus-responsive NDDSs for ALI

ALI is caused by various factors that lead to damage of alveolar epithelial cells and capillary endothelial cells, and is associated with uncontrolled inflammatory responses of the host immune system (Guo et al., 2021; Meyer et al., 2021). According to recent studies, early inhibition of pro-inflammatory cytokine release and the formation of ROS may be a promising therapy for ALI. Dexamethasone (Dex) was encapsulated into a poly (thioketone) polymer to form polymeric NPs (PTKNPs@Dex). In a ROS environment, the NPs can degrade responsively, releasing the drug and thereby reducing oxidative tissue damage (Zhai et al., 2022). Bilirubin (Br) and Atorvastatin (As) were encapsulated into the smart ROS-responsive nanocarrier DSPE-TK-PEG (DPTP), forming the NDDS (BA@DPTP). The high levels of ROS in ALI tissues trigger the drug release from BA@DPTP, thereby reducing ALI (Xia et al., 2024). A synergistic NDDS (AZI + IBF@NP) composed of ROS-responsive polymer (PFTU), antibiotics (Azithromycin, AZI), and anti-inflammatory drugs (Ibuprofen, IBF) was developed. ROS can be effectively cleared by this system in both *in vitro* and *in vivo* experiments (Muhammad et al., 2023). A nanosystem composed of oxidation-sensitive chitosan (Ox-CS), cerium nanoparticles (Ce NPs), and resatorvid (Ox-CS/CeRT NPs) has been developed. *In vitro* experiments have shown that Ox-CS/CeRT NPs can reduce ROS and inflammatory factors, thereby alleviating lung damage (Wu et al., 2023).

## 7 A clinical perspective on NDDSs for lung diseases

Currently, most approved or clinically developed nanomedicines are liposomal nanomedicines, polymeric nanomedicines, nanocrystal drugs, micellar nanomedicines, protein-based nanomedicines, or inorganic nanomedicines (Fernández-García and Fraguas-Sánchez, 2024). Nanocurcumin, prepared using nanotechnology, has shown positive effects in the treatment of various diseases (Yan et al., 2025). In a clinical study involving patients with COPD, nanocurcumin demonstrated an improvement in lung function (Zare’i et al., 2024). In another randomized controlled trial involving COVID-19 patients, nanocurcumin exhibited a positive effect on regulating inflammatory factors (Valizadeh et al., 2020). In LC patients, a recent clinical study has shown that nanoselenium significantly increases blood selenium levels in NSCLC patients and reduces the toxic side effects of chemotherapy (Song et al., 2021). In summary, NDDSs offer more hope for patients, but currently, the number of NDDSs entering clinical studies remains limited.

## 8 Discussion

With the continuous development of nanotechnology and polymer materials technology, research on stimulus-responsive strategies based on nanomaterials for pulmonary diseases has become increasingly in-depth. Due to the inherent ability of stimulus-responsive biomaterials to interact with the biological environment, they can be used as drug delivery carriers (Dou et al., 2020; Han et al., 2024; Wen et al., 2023). These “smart” biomaterials can respond to signals in the environment through mechanisms such as swelling/contraction, bond cleavage, surface changes, and structural changes. Drug release within NDDSs can be achieved through self-regulation or by direct or gradual activation via external or internal stimuli. By responding to specific stimulus signals, NDDSs can release drugs at the site of the lesion, ensuring that the highest concentration of the drug is achieved where it is most needed. This not only increases the local concentration of the drug but also prolongs its duration of action, thereby enhancing its efficacy. Additionally, this precise release of drugs under specific conditions can significantly reduce the required drug dosage (Li and Kataoka, 2021). Lower drug doses not only minimize toxic side effects but also lower treatment costs. Traditional drug delivery systems may require frequent dosing, whereas stimulus-responsive NDDSs can achieve prolonged drug release, reducing the frequency of patient dosing and thereby improving patient compliance. In summary, the development and application of stimulus-responsive NDDSs provide new approaches and methods for the treatment of lung diseases. By precisely controlling the timing and location of drug release, these “smart” biomaterials can significantly enhance drug efficacy, reduce toxicity, and improve patient compliance.

The advantages of endogenous stimulus-responsive NDDSs lie in their ability to utilize intrinsic stimuli within the body to regulate drug release, thereby achieving precise targeted therapy for diseased areas. This method reduces the need for external interventions, minimizes trauma to the body, and is more suitable for personalized medical strategies. However, endogenous stimulus-responsive NDDSs face challenges in precisely controlling their drug release behavior *in vivo,* and since not all internal stimuli are disease-specific, this can lead to unintended drug release in non-lesional areas. Additionally, different tumor types, physiological stages, and individual variations limit the clinical application of endogenous stimulus-responsive NDDSs. Currently, pH-responsive and enzyme-responsive NDDSs are widely studied, but these carriers still face difficulties in precisely controlling drug release at the lesion site and exhibit poor reproducibility, with few such drugs advancing to the clinical stage.

In contrast, the primary advantage of exogenous stimulus-responsive NDDSs is their remote controllability. By triggering drug release through external signals such as temperature, light, magnetic fields, and ultrasound, these carriers offer the potential for precise disease treatment. However, exogenous stimulus-responsive NDDSs also have some drawbacks. First, the spatiotemporal positioning of exogenous stimuli is difficult to accurately control, necessitating the introduction of localization components, which increases the complexity of carrier design. Second, the tissue penetration depth of exogenous stimuli also limits their applicability. Currently, more research is based on mouse models, which have limited *in vivo* validation. Furthermore, while some stimulus-responsive NDDSs have shown promising targeting effects in animal model experiments, delivering drugs precisely to the diseased area, they often exhibit off-target effects in clinical trials, thereby affecting treatment efficacy. The transition from basic research to clinical application requires further optimization of carrier design and functionality.

Despite many studies on stimulus-responsive delivery systems being reported, only a few of these systems have been tested *in vivo* in preclinical models, and even fewer have entered clinical phases. The biocompatibility, biodegradability, nontoxicity, and safer elimination from the biological system of stimulus-responsive carriers are important limitations that need to be considered before designing NDDSs. Different carrier materials can affect biocompatibility, and materials with good biocompatibility should be selected. For example, liposomes and polymer NPs are carrier materials that exhibit low toxicity and immunogenicity in the body. The microstructure and composition of nanomedicines are complex, and their construction process often involves multiple steps or sophisticated techniques, leading to poor reproducibility in preparation and making large-scale production extremely challenging. Additionally, the use of different analytical tools and testing methods by various research groups, coupled with the lack of standardized procedures for characterizing nanomedicine formulations, further complicates the research efforts. This inconsistency makes it difficult to conduct comprehensive comparative evaluations. Currently, the lack of animal models that accurately simulate human tumor conditions is a recognized deficiency in the field, resulting in weak correlations between preclinical studies and clinical trial outcomes (such as pharmacokinetics, biodistribution, and safety). Furthermore, regulatory frameworks struggle to keep pace with rapid technological advancements, creating a complex approval environment. Future research should focus on designing NDDSs with characteristics such as biocompatibility, biodegradability, and nontoxicity. To achieve better clinical translation, there is a need for more reproducible and scalable NDDSs and the development of *in vitro* and *in vivo* models that accurately reflect clinical characteristics. Novel equipment and technologies need to be accelerated and promoted to enable industrial-scale production of nanomedicines, ensuring they meet Good Manufacturing Practice (GMP) standards. Additionally, further improvements are needed in the evaluation systems for these drugs and the optimization of the approval processes for clinical trials.

## 9 Conclusion

Stimuli-responsive NDDSs have raised higher expectations for the treatment of lung diseases. Current research has focused more on animal models and has demonstrated their efficacy and safety, but lacks human trials. The preparation of economically viable and scalable nanomedicine systems is urgently needed. In conclusion, with the rapid development of nanomedicine, the continuous integration of basic and clinical research, and the collaborative efforts of researchers from various fields, these challenges are expected to be overcome. We believe that stimulus-responsive NDDSs for the treatment of pulmonary diseases will ultimately be successfully applied in clinical settings.
